# Flavonoids in mitigating the adverse effects of canine endotoxemia

**DOI:** 10.3389/fvets.2024.1396870

**Published:** 2024-08-13

**Authors:** Alma V. Móritz, Hédi Kovács, Ákos Jerzsele, Roland Psáder, Orsolya Farkas

**Affiliations:** ^1^Department of Pharmacology and Toxicology, University of Veterinary Medicine, Budapest, Hungary; ^2^National Laboratory of Infectious Animal Diseases, Antimicrobial Resistance, Veterinary Public Health and Food Chain Safety, University of Veterinary Medicine, Budapest, Hungary; ^3^Department of Internal Medicine, University of Veterinary Medicine, Budapest, Hungary

**Keywords:** canine, endotoxemia, cell culture, PBMC, PMN, flavonoids

## Abstract

In dogs, chronic enteropathies, and impaired gut integrity, as well as microbiome imbalances, are a major problem. These conditions may represent a continuous low endotoxin load, which may result in the development of diseases that are attributable to chronic inflammation. Flavonoids are polyphenolic plant compounds with numerous beneficial properties such as antioxidant, anti-inflammatory and antimicrobial effects. For our experiments, we isolated primary white blood cells (peripheral blood mononuclear cells and polymorphonuclear leukocytes) from healthy dogs and induced inflammation and oxidative stress with *Escherichia coli* and *Salmonella enterica* serovar Enteritidis lipopolysaccharide (LPS). In parallel, we treated the cell cultures with various flavonoids luteolin, quercetin and grape seed extract oligomeric proanthocyanidins (GSOP) alone and also in combination with LPS treatments. Then, changes in viability, reactive oxygen species (ROS) and tumor necrosis factor alpha (TNF-α) levels were measured in response to treatment with quercetin, luteolin and GSOP at 25 and 50 μg/mL concentrations. We found that ROS levels were significantly lower in groups which were treated by flavonoid and LPS at the same time compared to LPS-treated groups, whereas TNF-α levels were significantly reduced only by luteolin and quercetin treatment. In contrast, treatment with lower concentrations of GSOP caused an increase in TNF-α levels, while higher concentrations caused a significant decrease. These results suggest that the use of quercetin, luteolin and GSOP may be helpful in the management of chronic intestinal diseases in dogs with reduced intestinal barrier integrity or altered microbiome composition, or in the mitigation of chronic inflammatory processes maintained by endotoxemia. Further *in vitro* and *in vivo* studies are needed before clinical use.

## 1 Introduction

The use of natural plant-derived compounds as supplements to conventional medicine in small animal medicine is becoming increasingly popular. Flavonoids which belong to the polyphenol family, play an essential role in photosynthesizing cells (1), are found in many fruits and vegetables are well known for their positive health effect (2, 3). Flavonoids have a common benzo-γ-pyrone structure and can be subdivided into different subgroups depending on the side chain attached to it, such as flavonol, flavone, flavonone, flavanol, anthocyanidin and isoflavone subgroups. The flavonol subgroup includes quercetin, the flavone subgroup includes luteolin and the flavanol subgroup includes proanthocyanidins among many other molecules (1). The biological and pharmacokinetic properties of flavonoids are also an area of extensive research. Flavonoids have been used to reduce oxidative stress and systemic inflammation (4). These compounds act as electron acceptors to bind oxygen and nitrogen containing free radicals, inhibit the effects of pro-oxidants (5, 6) and protect cells (7). Flavonoids also affect immune cells, the production of cytokines and other pro-and anti-inflammatory proteins (8), inhibit the activation of major cell signaling pathways responsible for inflammation (5), including toll-like receptors, nuclear factor kappa-light-chain-enhancer of activated B cell pathways (9), as well as Kelch-like ECH-associated protein-1 activation and nuclear factor erythroid 2-related factor 2 nuclear translocation (10). These compounds also affect enzyme activities, such a cyclooxygenase-2, lipoxygenase-2 (9, 10), as well as nitric oxide synthase, mitogen activated protein kinase and protein kinase-C (10). In addition, flavonoids also play a role in infections by reducing inflammation in infections, influencing host-pathogen interactions and immune responses, and reducing viral load and the deleterious effects of the cytokine storm resulting from viral infection (4), among others in COVID-19 infection (11, 12). Some representatives of flavonoids have prebiotic-like effects on the gut microbiome, strengthening the intestinal barrier and thereby reducing chronic inflammatory processes and glycolipid-related metabolic disorders (13). Flavonoids may be important players in the prevention and treatment of diseases such as diabetes, obesity, metabolic syndrome (2) or inflammatory bowel disease (IBD) (10).

Quercetin is a flavonoid that has long been used as part of Chinese medicine. It can be obtained as part of the daily diet from vegetables, fruits, and even tea (7, 14, 15). Quercetin is well known for its antioxidant, antimicrobial, anti-inflammatory, antiviral, antifungal and anticancer effects. It reduces the proliferation of tumor cells and stimulates their apoptosis (7). It has been shown to have protective effects in cerebellar ischemia and to strengthen the blood–brain barrier (14) and may also be beneficial in the adjuvant treatment of cardiovascular diseases by reducing cholesterol levels, improving endothelial function, and lowering blood pressure (7, 16). Luteolin is a yellow-colored flavonoid (17) first isolated from *Reseda luteola* but is also found in several fruits and vegetables, including carrots, apples, peppers, olive oil and herbs (18). Luteolin also possesses antioxidant, anti-inflammatory, cardioprotective and neuroprotective effects (19, 20), and thus its beneficial effects may help in Alzheimer’s-like disease and diseases with a gut microbiota-liver-brain axis background (17). It is known for its antitumor activity (19, 21, 22), antibacterial (16) and anti-allergic effects (23). Due to its anti-allergic activity, it may play a role in the additional therapy of asthma or allergic skin diseases such as atopic dermatitis (23, 24). Proanthocyanidins are also very common active polyphenolic compounds. They are found in grapes, apples, grapefruit, berries, pine bark and cocoa. They are also called as condensed tannins; they form oligomers and polymers flavanes with single or double bonds (25). Grape seed extract proanthocyanidins (GSOP) also have anti-inflammatory (26) antioxidant (27), anticarcinogenic, antibacterial effects (28, 29), and beneficial properties in osteoarthritis was also described (3). Proanthocyanidins reduce the concentration of free radicals and inhibit their release. The large number of hydroxyl groups in proanthocyanidins also contributes to their antimicrobial activity. They prevent bacterial adhesion and aggregation, reduce biofilm formation and reduce inflammation. Their proven antimicrobial activity against *Escherichia coli* (*E. coli*) have been also described (30).

The largest part of the digestive system is the small intestine, which has an enormous surface area in contact with the outside environment. Antigens, allergens, toxins and pathogenic microbes can enter the body through food or food intake (31) and can trigger immune tolerance or immune responses. In addition to digesting and absorbing food, the intestinal tract plays an important role in the defense against exogenous pathogens due to its specific histology and its close relationship with the immune system (32). The metabolism of natural compounds of plant origin that enter the body starts in the gut. Flavonoids are usually present in plants in the form of glucosides and are converted to aglycones during hydrolysis in the small intestine (33). Quercetin is able to undergo glucuronidation, sulfation and methylation in enterocytes and hepatocytes due to hydroxyl groups in its structure (34), thus converting it into less biologically active metabolites. Intestinal microbiota also affects the absorption and metabolism of quercetin, further complicating bioavailability, and its poor solubility in water also affects its absorption. It has been observed that quercetin glucosides had the highest rate of absorption from the intestinal tract (7). Luteolin exerts anti-inflammatory effects in the gut by reducing the production of inflammatory cytokines such as interleukin-1β (IL-1β), interleukin-6 (IL-6), and interferon-β (INF-β) (33) and by reducing the activity of TNF-α stimulated by lipopolysaccharides, which is involved in the development of the body’s inflammatory response (10). After ingestion luteolin can be converted while passing through the intestinal mucosa and absorbs in a monoglucuronide form or it can reach the blood plasma without transformation (35). The proanthocyanidins are absorbed from the intestinal tract by passive diffusion and are then returned to the small intestine by enterohepatic recirculation. Proanthocyanidins are distributed to different parts of the body such as the lungs, kidneys and spleen, but most go to the large intestine and may provide protection by various mechanisms such as anti-inflammatory and antioxidant (30), making them a potential adjunct agent in the treatment of inflammatory bowel disease.

Chronic enteropathies are common complex diseases in dogs (36). One form of chronic inflammatory enteropathy (CIE) affects nearly one in five dogs (37). Canine chronic inflammatory enteropathy (CIE) is a fairly common problem that affects the quality of life of both the animal and the owner (38). Typically, it is a condition with vomiting, diarrhea, worsening or recurrent complaints lasting more than 3 weeks and the diagnosis can be made by various additional tests (fecal examination, abdominal ultrasound, gastroscopy, colonoscopy, histopathology) (39). Before their diagnosis, extraintestinal origin, chronic parasitic and bacterial intestinal infections, intestinal obstruction and intestinal tumors should be excluded (40). Their development may be due to damage to the local immune system, possibly caused by an abnormal immune response, but also feeding and diet significantly influence, e.g., fat intake (41, 42) or overweight (43–45), the health of the intestinal tract. It could be influenced directly through intestinal stem cells or epithelial integrity and also indirectly through the microbiome (46–48). Genetic predisposition may also play a role, and previous viral, bacterial or parasitic infections and antibiotic treatment may act as a factor increasing the permeability of the intestinal mucosa and damaging the microbiota. The resulting allergic and inflammatory processes can lead to food-responsive enteropathy (FRE), antibiotic-responsive enteropathy (ARE), immunosuppressant-responsive enteropathy (IRE) and non-responsive enteropathy (NRE) forms of CIE. The FRE form is the most common, accounting for nearly two-thirds of CIE. ARE may be caused by dysbiosis or bacterial overgrowth in the small intestine. The gastrointestinal microbiota of dogs is a unique and stable ecosystem (49), formed by 10^12^–10^14^ microbes (50). The composition of the canine microbiota is more similar to that of humans than that of pigs or mice (51), with the majority of microbes belonging to the Firmicutes, Fusobacteria, Bacteroidetes and Proteobacteria phyla (52, 53). Alterations in the composition of the microbiome lead to dysbiosis. In dysbiosis, the biodiversity of the microbiome decreases, the number of Firmicutes, Bacteriodes, Clostridales strains decreases, including the bacterium *Clostridium hiranonis*, which plays an important role in intestinal bile acid metabolism, while the proportion of Proteobacteria increases (49, 52, 53). The IRE and NRE group also includes idiopathic inflammatory bowel disease (IBD), which etiology is unknown (54) and requires histopathological examination of the intestinal mucosa to determine it. However, it is reported to be a multifactorial disease, involving intestinal mucosal immunity, environmental factors, the microbiome and nutrition and genetics of the animal (10). Pathophysiologically, IBD is most often associated with lymphocytic-plasmacytic enteritis (55), eosinophilic enteritis, eosinophilic gastritis, neutrophilic enteritis suspected to be caused by *Salmonella* or *Campylobacter* infection, and granulomatous enteritis. IBD is most common in German Shepherd, Shar-Pei and Basenji dogs, but can also occur in cats, often in association with pancreatitis and biliary tract inflammation. Malabsorption due to intestinal inflammation can result in hypoproteinaemia. Sequencing of fecal samples from the feces suggests that *E. coli* infection may be the cause of IBD (56), and that a reduction in *Clostridium hiranonis* may also play an important role in the development of the disease. The anatomical and physiological similarity of the intestinal tract of dogs and humans, and the similarity of dog-human diet and daily rhythms, make dogs a good model animal for the multifactorial and complex human disease IBD (57, 58).

IBD is associated with oxidative stress, and the inflammatory cytokines released during this process increase the production of ROS and inflammation (10). The disease may also pose a risk of endotoxemia, as damage of the intestinal epithelium may result in the intestinal cell wall-forming toxins of the bacteria in the gut being more easily released into the circulation, and these bacterial endotoxins (lipopolysaccharides, LPS) may increase the concentration of specific inflammatory markers in the body (38). Digestive tract problems are a common pathology in dogs and can result in endotoxemia (59) caused by lipopolysaccharides (38). The intestinal epithelium has a barrier role, preventing the absorption of lipopolysaccharides. Dysbiosis can cause elevated levels of intestinal lipopolysaccharides (LPS), which can trigger local inflammatory processes, with activation of toll-like receptors (TLR) and pathogen associated molecular patterns (PAMPs) (60). Dysbiosis also can increase intestinal permeability via injuries and barrier function impairment. These resulting in the release of pathogenic bacteria and bacterial constituents such as LPS into the bloodstream which lead to metabolic and immunological imbalances and chronic low inflammation (61–63), in consequences metabolic or immune diseases such as obesity, diabetes (64), hepatic lipidosis, IBD, osteoarthritis, asthma, myasthenia gravis (61), tumors (65), cardiovascular (38), and neuropathological diseases can occur (66). LPS affect the production of pro-and anti-inflammatory cytokines and TNF-α (26) besides chronic low LPS load increases A20 exposure, which positively regulates PPAR-α and-γ, thus dampens the NF-κB signaling pathway and NLRP3 inflammasome activation (67). LPS affects nitric oxide synthetase activity and increases reactive nitrogen species and reactive oxygen species (ROS) (26). LPS induces neutrophil granulocyte cell dysfunction, which may lead to tissue damage. The dysfunction of neutrophils may also result in impairment of adhesion, phagocytosis, chemotaxis, and intracellular bactericidal function of monocytes (68). These complex pathological processes may result in the development of diseases that are attributable to chronic inflammation (62). It is not proven whether dysbiosis is the triggering cause or a consequence of diseases associated with dysbiosis (69).

Traditionally, CIE can be managed by dietary feeding, medication and probiotics, and also endotoxemia can often be prevented with probiotics (59). CIE can be well managed in most cases by establishing a suitable diet for the dog, but not always effectively (37, 70). There are cases where other means such as glucocorticoids and immunosuppressive agents may be needed. Immunosuppressive agents such as prednisolone (55) are used in treatment, but these have a number of side effects. Polyphagia, polyuria, polydipsia and changes in the animal’s behavior have been often observed. In the long term, overweight, muscular atrophy and weakness, urinary tract infections and even diabetes may occur (54). Because of the above mentioned it is worth looking for complementary agents that can help to reduce the dose of immunosuppressive drugs and reduce the systemic endotoxin load (38). Since flavonoids are well-known about their anti-inflammatory and antioxidant properties so they can be an important therapeutic option in these conditions.

In the present study we modeled the role of the flavonoids in endotoxemia therefore the anti-inflammatory and antioxidant effects of quercetin, luteolin and GSOP were investigated in primary isolated canine white blood cell cultures challenged by bacterial LPS.

## 2 Materials and methods

### 2.1 Sample collection

These experiments were performed on peripheral blood mononuclear cells (PBMC) and polymorphonuclear leukocytes (PMN) cultures. Blood for *in vitro* PBMC and PMN cell cultures was obtained with the owner’s consent from a healthy regularly vaccinated and dewormed dog during yearly monitoring without any symptoms. Animal procedures were performed according to the international and national law as well as the institutional guidelines and were confirmed by the Government Office of Pest County, Food Chain Safety, Plant Protection and Soil Conservation Directorate, Budapest, Hungary (permission number: PE/EA/00980–6/2022). Blood was collected in EDTA tubes and white blood cell isolation was started within 30 min after blood collection.

### 2.2 PBMC and PMN isolation and culture conditions

Two different densities of media were used to separate the two fractions of white blood cells from other fractions. Histopaque 1.077 (Sigma-Aldrich, St. Louis, United States) and Histopaque 1.119 (Sigma-Aldrich, St. Louis, United States) were used for isolation according to the manufacturer’s instructions. Three milliliter of Histopaque 1.077 solution was pipetted into a centrifuge tube and 3 mL of Histopaque 1.119 solution was added, then 6 mL of coagulated whole blood was put on top. Centrifugation was performed at 700 g for 30 min at room temperature. After centrifugation, the two white blood cells rich separated fractions, the PBMC and the PMN, were collected one by one in new centrifuge tubes. The separated fractions were added 1 mL RBC lysis solution (Roche Diagnostics, Mannheim, Germany) and were gently swirled for 1 min. After that they were supplemented with 1x phosphate buffered saline (PBS Gibco, Paisley, United Kingdom) to reach the final volume of 10 mL and centrifuged at 400 g for 7 min at room temperature. The process was repeated until the fractions were completely cleaned of red blood cells. Then we washed the fractions with 10 mL PBS twice and centrifuged them at 200 g for 10 min at room temperature (71). Finally, the number of living cells was determined by Bürker chamber counting using trypan blue (Sigma-Aldrich, St. Louis, United States) staining. The cells were seeded onto different cell culture plates at a density of 2 × 10^5^ cells/mL. The PBMC and PMN cells were cultured in RPMI-1640 medium with L-glutamate and sodium bicarbonate (Sigma-Aldrich, St. Louis, United States) supplemented with 10% fetal bovine serum (EuroClone, Pero, Italy) and 1% PenStrep (Lonza, Verviers, Belgium). Cells were cultured on 24 and 96-well plates. They were incubated overnight at 37°C in a thermostat containing 5% CO_2_ and 95% humidity.

After 24 h incubation, to induce oxidative stress and inflammation, *Escherichia coli* O111:B4 (Sigma-Aldrich, St. Louis, United States) and *Salmonella enterica* serotype Enteritidis (Sigma-Aldrich, St. Louis, United States) LPS were applied at concentrations of 0.1 μg/mL, 1 μg/mL, and 10 μg/mL. Among the flavonoids, quercetin (≥95%, Sigma-Aldrich, India), luteolin (≥98%, Sigma-Aldrich, Israel), purified GSOP (≥98.8, USP, Rockville) were used for our experiments at concentrations of 12.5; 25; 50 μg/mL to monitor their antioxidant and anti-inflammatory potential. The solutions were prepared with plain RPMI-1640 medium and were applied to cells after supplemented medium had been drained. The chosen concentrations were based on our previous studies and were in line with the used concentrations in the literature for *in vitro* experiments (72, 73). Samples were taken 24 h after treatment.

The control group was treated with medium only, and there were only LPS-treated and flavonoid-treated groups, which results were compared to the control groups. In addition, LPS treatments were combined with flavonoid treatments, which results were compared to the only LPS-treated groups. Six to eight replicates were tested for each group.

### 2.3 Determination of the antioxidant effect of the investigated flavonoids

To test the antioxidant effect of flavonoids, their radical scavenging capacity was verified by the DPPH assay (Dojindo EU, Munich, Germany). DPPH (2,2-Diphenyl-1-picrylhydrazyl) is a stable free radical compound. The odd electron of nitrogen atom in DPPH is reduced by receiving a hydrogen atom from antioxidants to the corresponding hydrazine (74). The assay was performed following the manufacturer’s guidance. Absorbance was measured at 517 nm, using Spectramax iD3 (Molecular Devices, San Jose, CA, United States) (75). The IC_50_ values for the radical scavenging capacity of antioxidants were calculated based on the obtained absorbance values, following the instructions of the manufacturer.

### 2.4 Cell viability measurements with cell counting kit-8 assay

Cell counting kit-8 assay (CCK-8, Sigma-Aldrich, Japan) was performed to test the metabolic activity of cells and to monitor the effects of LPS and flavonoids on cell metabolic processes. CCK-8 is a sensitive colorimetric assay. It contains WST-8 a tetrazolium salt, which is reduced to formazan in the presence of an electron mediator. The method was applied according to the manufacturer’s instructions. In a 96-well plate, 10 μL of CCK-8 solution was added to treated PBMC and PMN cells and after 2 h of incubation at 37°C, 5% CO_2_, absorbance was measured at 450 nm using Spectramax iD3 (Molecular Devices, San Jose, CA, United States).

### 2.5 Protein measure and cell counting

Considering the possibility that samples contained different amounts of cells, the BCA Protein Assay Kit (ThermoFisher, Kandel, Germany) was performed. The assay was carried out according to the manufacturer’s instructions. Absorbance was measured after 30 min incubation at 562 nm with Spectramax iD3 (Molecular Devices, San Jose, CA, United States). Protein amount of samples were calculated from the absorbance values, measurement results have been corrected for protein content.

### 2.6 Determination of the amount of intracellular reactive oxygen species

2′,7′-dichlorofluorescein-diacetate (DCFH-DA, Sigma-Aldrich, Israel) is a non-fluorescent compound that can be used to non-specifically measure the amount of ROS produced intracellularly. DCFH-DA undergoes a molecular transformation in the presence of ROS, losing its acetate group and converting to the fluorescent 2′,7′-dichlorofluorescein. The intensity of the measured fluorescence is therefore proportional to the amount of ROS present in the cells. DCFH-DA is light sensitive and was therefore used in the dark. For DCFH-DA 40 mM stock solution, 5 mg of powder was measured and dissolved in dimethyl sulfoxide (Sigma-Aldrich, Darmstadt, Germany). A 4 μM treatment solution was prepared from our stock solution by adding plain DMEM/F12 nutrient mixture (Gibco, Paisley, United Kingdom). After PBS washing, we treated our cultures with 0.5 mL/well of treatment solution and incubated at 37°C for 60 min. After incubation, Mammalian Protein Extraction Reagent (M-PER, ThermoFisher, Rockford, United States) was added to the cells (200 μL/well), shaken for 5 min, dissolved cells were pipetted into Eppendorf tubes, centrifuged for 10 min at 4,500 RPM at 4°C and finally 100–100 μL of the supernatants were pipetted into 96-well plate. Fluorescence intensity was then measured at 480 nm excitation, 530 nm emission wavelength using Spectramax iD3 (Molecular Devices, San Jose, CA, United States).

### 2.7 Measurements of TNF-α levels

The effect of LPS of different origin and flavonoids on TNF-α production was tested on the PBMC fraction. For our assays, we used canine TNF-α sandwich ELISA kit according to the manufacturer’s instructions (Sigma-Aldrich, Germany). Samples were taken from the cell supernatant at 24 h after treatments and TNF-α concentrations were determined. Absorbance measurements were performed at 450 nm. Calibration was performed during the assay and that the absorbance values were converted to pg/mL concentrations.

### 2.8 Statistical analysis

Statistical calculations were performed using R 3.3.2 (2016) software (R Foundation, Vienna, Austria). Diagnostic analysis was used to exclude bias points and to determine the normal distribution of residuals. One-way analysis of variance (ANOVA) using the *post hoc* Tukey test for multiple comparisons was performed. The significance level was set at 5% (*p* < 0.05) when testing for differences between groups. *p* < 0.05, *p* < 0.01, and *p* < 0.001 indicate statistically significant differences.

## 3 Results

### 3.1 Determination of the antioxidant effect and cell viability measurement

Using the DPPH assay, the IC_50_ values of flavonoid compounds were determined. The IC_50_ value for quercetin was 24.8 μg/mL, while luteolin was 49.4 μg/mL and GSOP was 48.3 μg/mL.

**Figure 1:**
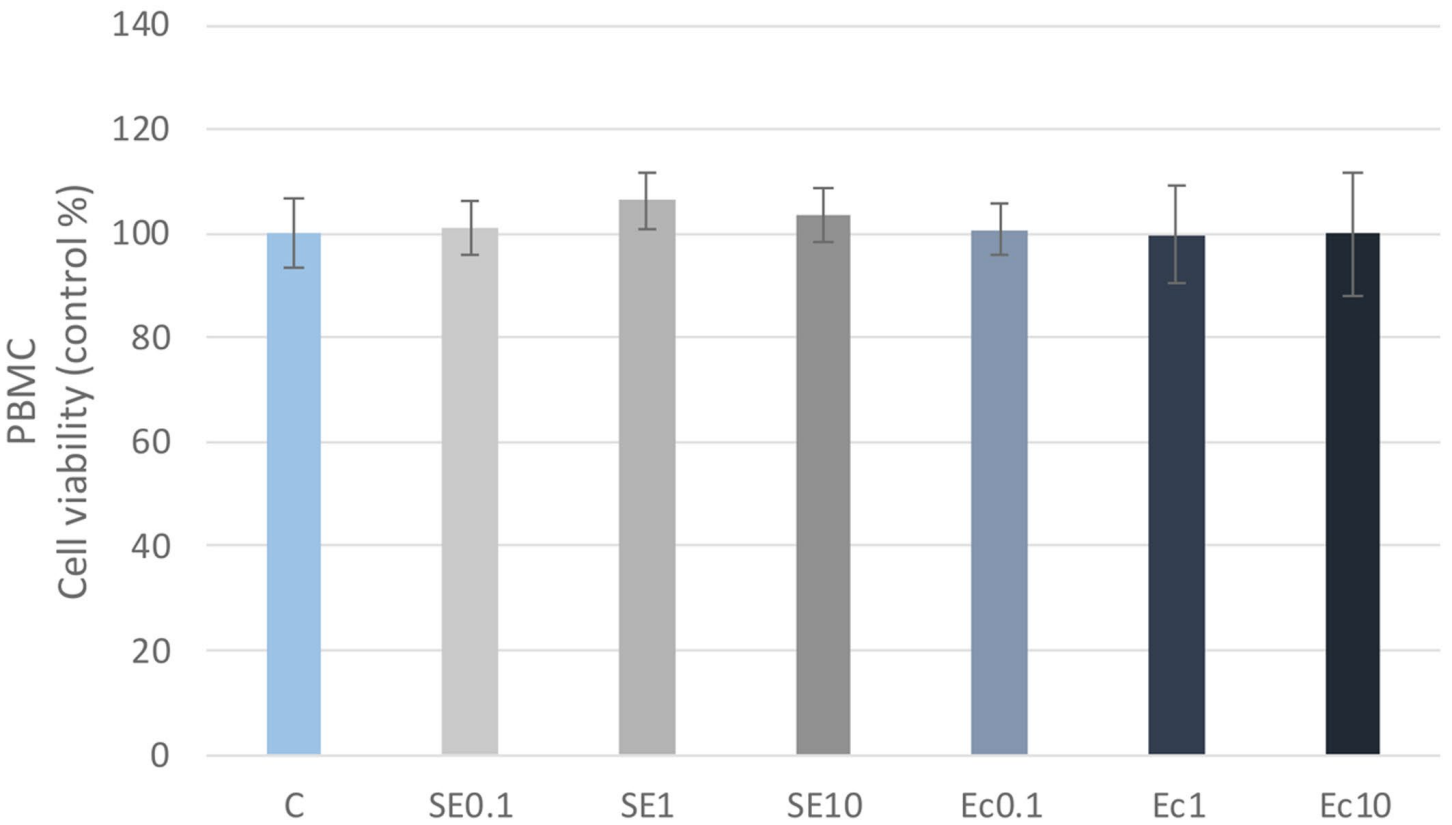
Investigation of LPS treatment on cell viability in PBMC culture with CCK-8 assay. The results are shown as mean values, along with their corresponding standard deviations. Mean of the control group set at 100%, (*n* = 8/group). C: control, SE0.1, SE1, SE10: *S. enteritidis* LPS 0.1, 1, 10 μg/mL, Ec0.1, Ec1, Ec10: *E. coli* LPS 0.1, 1, 10 μg/mL.

**Figure 2:**
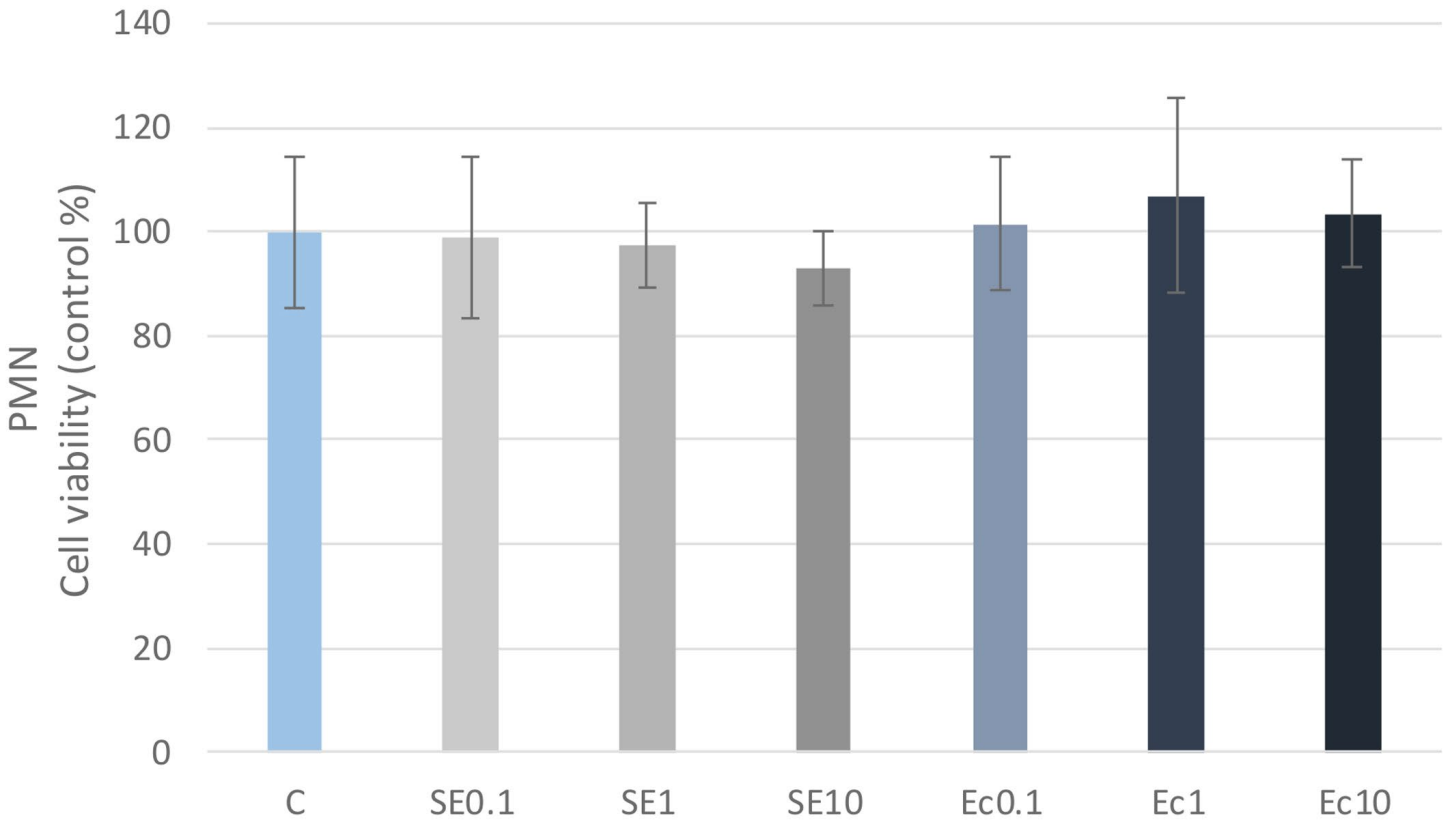
Investigation of LPS treatment on cell viability in PMN culture with CCK-8 assay. The results are shown as mean values, along with their corresponding standard deviations. Mean of the control group set at 100%, (*n* = 8/group). C: control, SE0.1, SE1, SE10: *S. enteritidis* LPS 0.1, 1, 10 μg/mL, Ec0.1, Ec1, Ec10: *E. coli* LPS 0.1, 1, 10 μg/mL.

No significant differences in cellular metabolic activity were observed in the three concentrations of the *E. coli* and *S. enteritidis* LPS treatment in either PBMC or PMN cell cultures compared to the control group (Figures 1, 2). Absorbance values were converted to control percentages.

**Figure 3:**
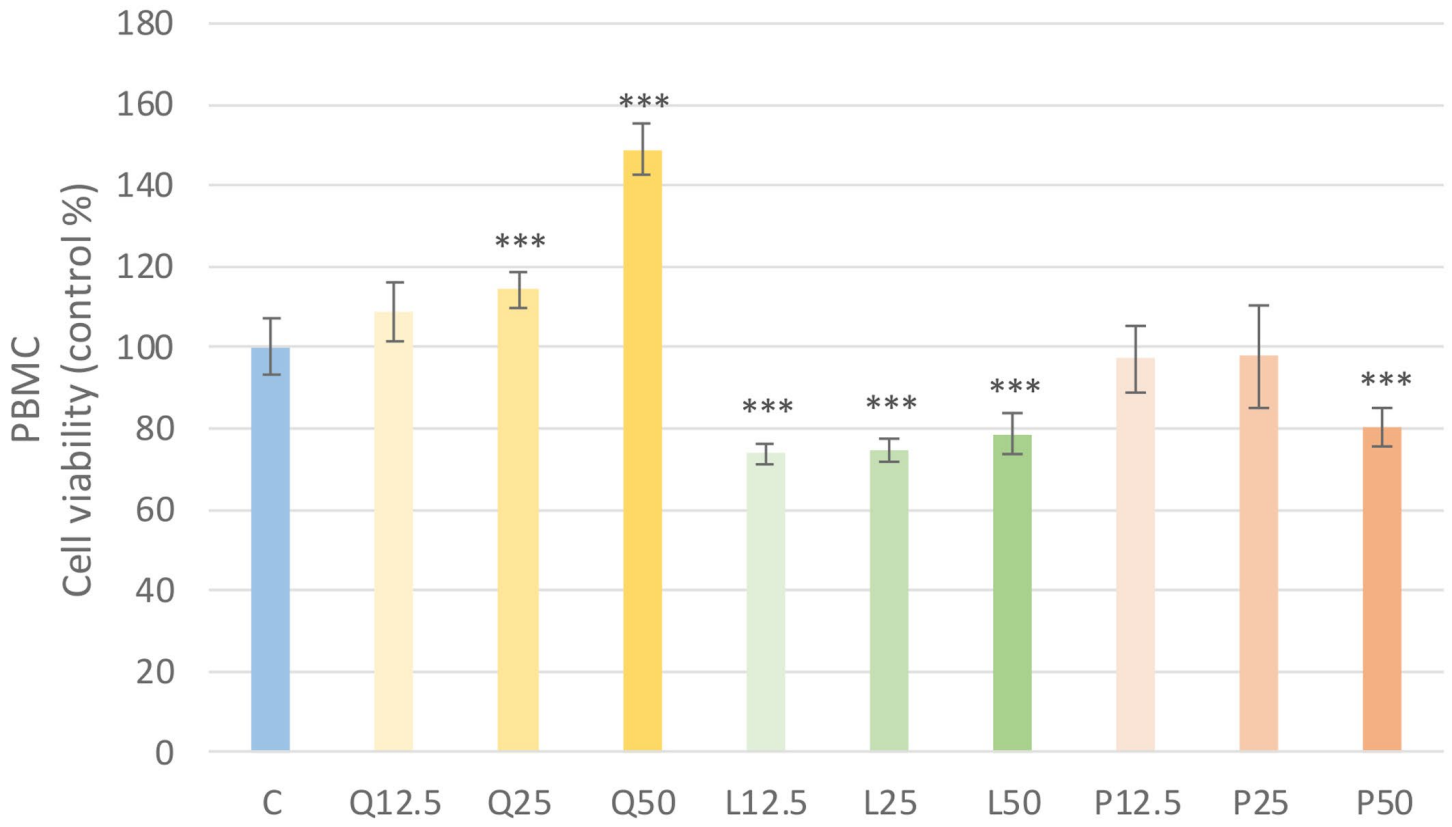
Investigation of quercetin, luteolin and GSOP treatment on cell viability in PBMC culture with CCK-8 assay. The results are shown as mean values, along with their corresponding standard deviations. Mean of the control group set at 100%, (*n* = 8/group). C: control, Q12.5, Q25, Q50: quercetin 12.5, 25, 50 μg/mL, L12.5, L25, L50: luteolin 12.5, 25, 50 μg/mL, P12.5, P25, P50: GSOP 12.5, 25, 50 μg/mL. Significant difference: ***p < 0.001 compared to control group.

**Figure 4:**
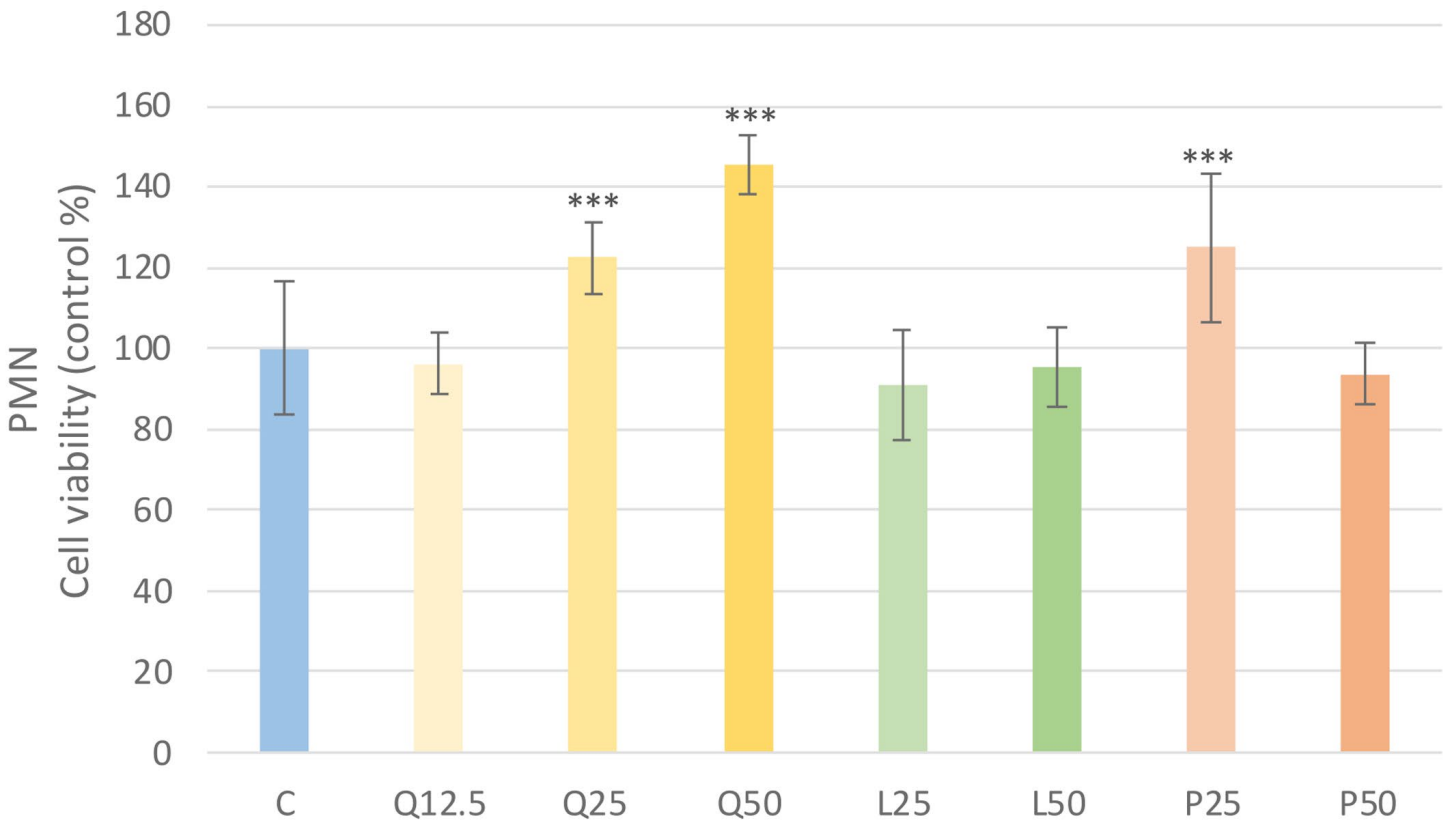
Investigation of quercetin, luteolin and GSOP treatment on cell viability in PMN culture with CCK-8 assay. The results are shown as mean values, along with their corresponding standard deviations. Mean of the control group set at 100%, (n = 8/group). C: control, Q12.5, Q25, Q50: quercetin 12.5, 25, 50 μg/mL, L12.5, L25, L50: luteolin 12.5, 25, 50 μg/mL, P12.5, P25, P50: GSOP 12.5, 25, 50 μg/mL. Significant difference: ***p < 0.001 compared to control group.

The metabolic activity of the cells was tested after treatment with three different concentrations of the flavonoids. Absorbance values were converted to control percentages and compared to the control group in PBMC and PMN cultures. Both PBMC and PMN cultures showed a significant increase (*p* < 0.001) in metabolic intensity of quercetin treatment at 25 and 50 μg/mL concentrations, while luteolin at all three concentrations and GSOP at 50 μg/mL concentration resulted in a significant decrease (*p* < 0.001) in metabolic activity in PBMC cells compared to the control group. In contrast, in PMN cells, luteolin did not cause a significant difference, whereas GSOP at 25 μg/mL resulted in a significant increase (*p* < 0.001) in metabolic activity compared to the metabolic activity of cells in the control group (Figures 3, 4).

### 3.2 Determination of the amount of intracellular reactive oxygen species

**Figure 5:**
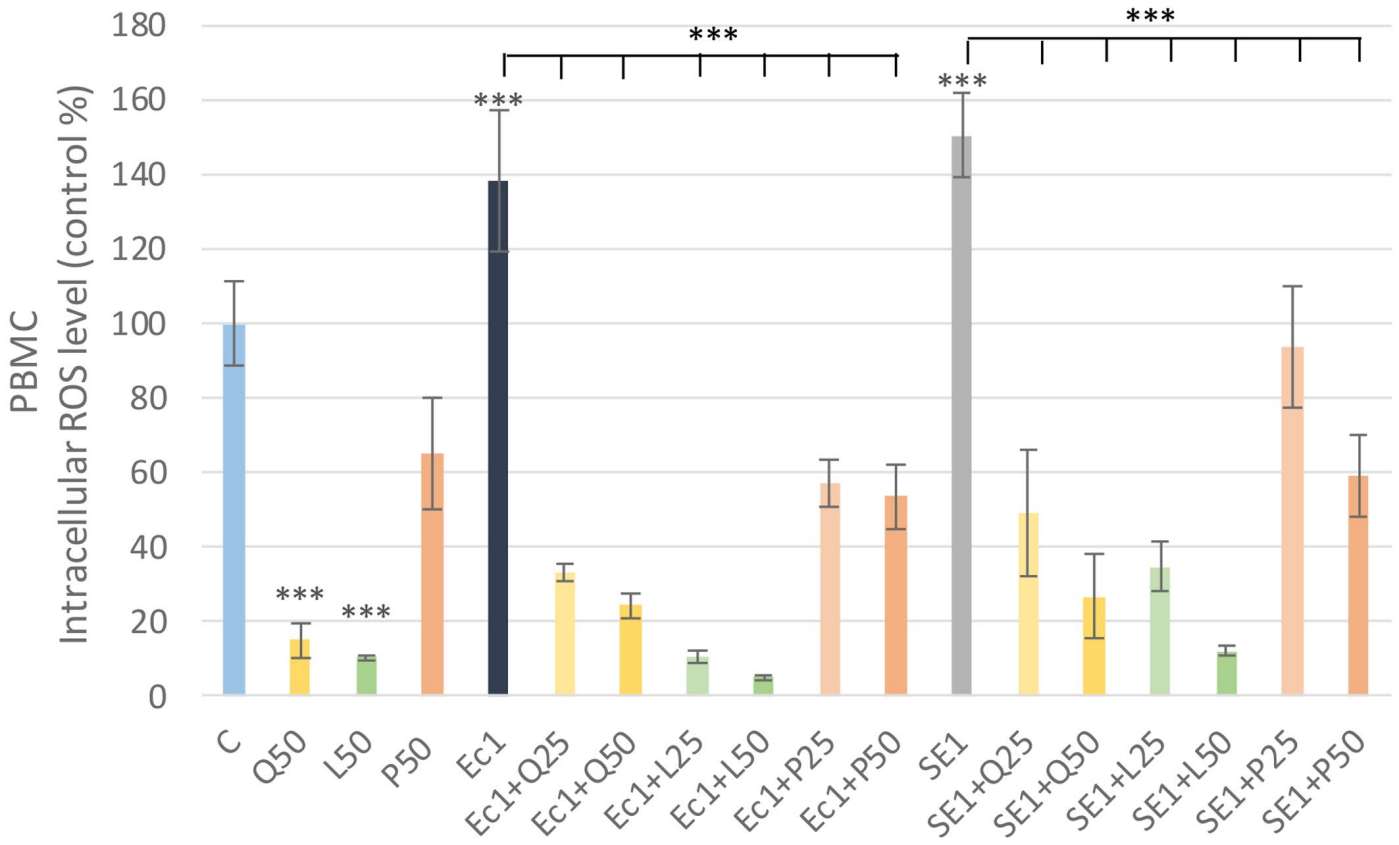
Investigation of quercetin, luteolin and GSOP treatment on ROS levels in PBMC culture with DCFH-DA assay. The results are shown as mean values, along with their corresponding standard deviations. Mean of the control group set at 100%, (*n* = 6/group). C: control, Q25, Q50: quercetin 25, 50 μg/mL, L25, L50: luteolin 25, 50 μg/mL, P25, P50: GSOP 25, 50 μg/mL, Ec1: *E. coli* LPS 1 μg/mL, SE1: *S. enteritidis* LPS 1 μg/mL. Significant difference: ***p < 0.001 compared to control group or LPS treated groups.

LPS derived from *E. coli* and *S. enteritidis* in 1 μg/mL caused a significant increase (*p* < 0.001) in intracellular ROS level compared to the control group in PBMC. Among the investigated flavonoids, quercetin and luteolin alone caused a significant decrease (*p* < 0.001) in the intracellular ROS amount compared to the control group. The LPS and flavonoid treatments combinations showed a significant decrease (*p* < 0.001) in intracellular ROS level compared to the LPS-treated groups. Fluorescence intensity values were converted to control percentages (Figure 5).

**Figure 6:**
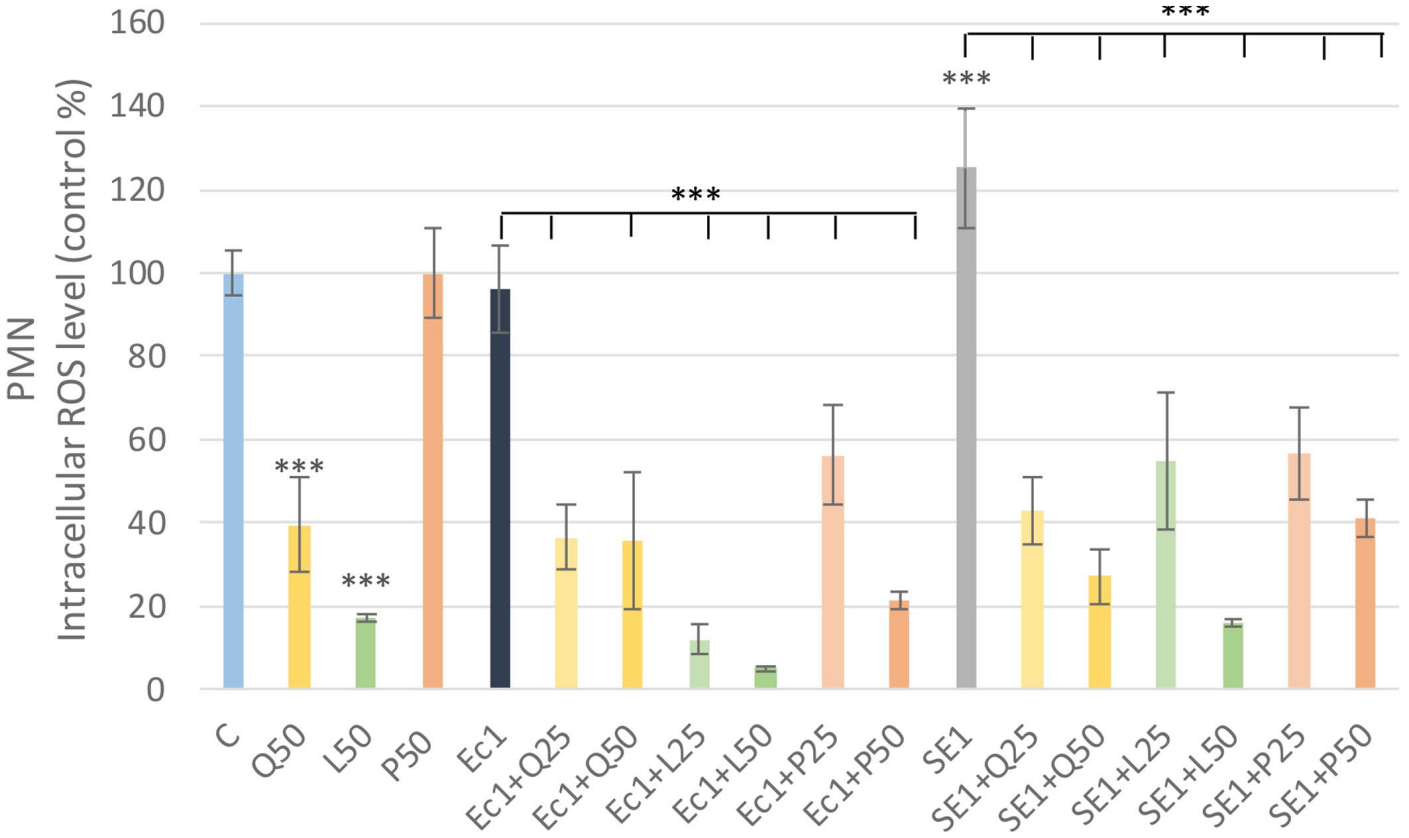
Investigation of quercetin, luteolin and GSOP treatment on ROS levels in PMN culture with DCFH-DA assay. The results are shown as mean values, along with their corresponding standard deviations. Mean of the control group set at 100%, (*n* = 6/group). C: control, Q25, Q50: quercetin 25, 50 μg/mL, L25, L50: luteolin 25, 50 μg/mL, P25, P50: GSOP 25, 50 μg/mL, Ec1: *E. coli* LPS 1 μg/mL, SE1: *S. enteritidis* LPS 1 μg/mL. Significant difference: ***p < 0.001 compared to control group or LPS treated groups.

Quercetin and luteolin alone also caused a significant decrease (*p* < 0.001) compared to the control group, but no significant increase was observed in PMN after LPS treatment compared to the control, however, the addition of flavonoids resulted in a significant decrease (*p* < 0.001) in ROS in all LPS + flavonoid treated groups. Fluorescence intensity values were converted to control percentages (Figure 6).

### 3.3 Measurements of TNF-α levels

**Figure 7:**
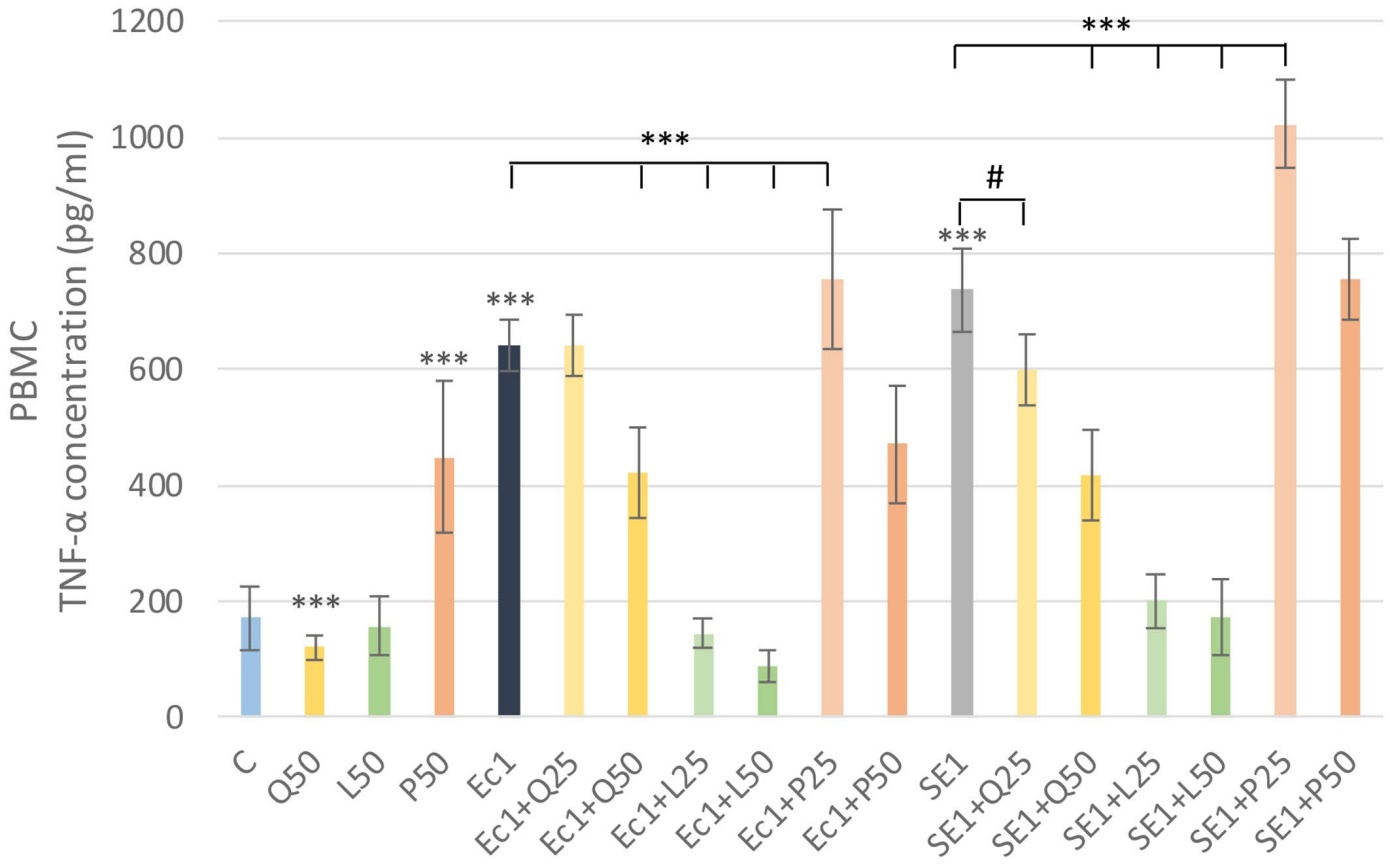
Investigation of quercetin, luteolin and GSOP treatment on TNF-α levels in PBMC culture with ELISA. The results are shown as mean values, along with their corresponding standard deviations. Mean of the groups are in TNF-α concentrations (pg/mL), (*n* = 6/group). C: control, Q25, Q50: quercetin 25, 50 μg/mL, L25, L50: luteolin 25, 50 μg/mL, GSOP 25, 50 μg/mL, Ec1: *E. coli* LPS 1 μg/mL, SE1: *S. enteritidis* LPS 1 μg/mL. Significant difference: #*p* < 0.1 compared to SE LPS treated group, ***p < 0.001 compared to control group or LPS treated groups.

In the analysis of TNF-α levels, LPS treatments caused a significant increase (*p* < 0.001) compare to the control group. Quercetin significantly reduced (*p* < 0.001) the TNF-α increase induced by *S. enteritidis* LPS at a concentration of 50 μg/mL. Luteolin significantly reduced (*p* < 0.001) TNF-α levels for both LPS at both concentrations. In contrast, the addition of GSOP at a concentration of 25 μg/mL resulted in a significant increase (*p* < 0.001) for both LPS (Figure 7). We also investigated TNF-α levels in PMN cells, but the measured absorbance values did not differ from the control values for either treatment.

## 4 Discussion

An increasing number of systemic diseases are thought to be caused by damage to the gut barrier and decrease of the diversity of the gut microbiome (61). Microbiome is a significant modulator of the immune system (76). Dogs have a similar gut and microbiome architecture to humans and have similar chronic intestinal diseases (51). Changes in the composition of the microbiome can have serious systemic consequences. There are some chronic inflammatory conditions which can correlated with changes in microbiome for example IBD, obesities and diabetes. Changes in the composition of the microbiome result in a decrease in Firmicutes and an increase in Proteobacteria in IBD (77).

Many plants and plant extracts have been used for thousands of years by humans for their medicinal properties, among others mitigate gastroenterological disorders. More than 40 different medicinal plants are used in the European Union for treatment of gastrointestinal diseases based on traditional use, mainly for treatment of chronic gastrointestinal disorders. These plants have gastric secretion stimulating, antispasmodic, intestinal musical soothing, laxative, anti-diarrheal and anti-inflammatory effects (78). However, it is poorly understood which compounds or combinations of compounds in plants are responsible for some of these effects. Because of the similarities in the intestine of humans and dogs may make the findings more easily adaptable to dogs. Flavonoids are commonly used polyphenolic plant compounds with many beneficial effects. They have attracted much attention in both human and veterinary medicine for their health-enhancing and disease-preventive effects. They are also known for their antioxidant, anti-inflammatory, antimicrobial and anticancer activities, as well as their free radical scavenging ability (1). Flavonoids inhibit the action of a number of enzymes in the body, such as cyclooxygenase, lipoxygenase, xanthine oxidase and aldose reductase (79). These enzymes contribute to the development of inflammation, and their inhibition can therefore achieve anti-inflammatory activity during both the proliferative and exudative phases of inflammation (80). The antioxidant, anti-inflammatory and antimicrobial effects of quercetin, luteolin and proanthocyanidins are well known. In dogs, chronic intestinal inflammation and impaired gut integrity, as well as microbiome imbalance, are a major problem. These conditions may represent a continuous low endotoxin load. Flavonoids can play an important role in mitigating the adverse effects of endotoxemia. The aim of our research was to demonstrate the antioxidant and anti-inflammatory effects of flavonoids in *in vitro* canine white blood cell cultures.

To induce inflammation and oxidative stress LPS was used. LPS is a glycolipid, has a hydrophobic lipid part and a hydrophilic polysaccharide part. These LPS molecules are linked together to form a tight hydrophobic structure with strong bonds, reducing membrane permeability, which allows, for example, protection against antimicrobial factors (81). LPSs are the most abundant endotoxin molecules, which have an inflammation-stimulating effect when circulating in the blood. They are a major component of the outer cell membrane of Gram-negative bacteria, which are found in large numbers in the intestinal tract. These bacteria can induce inflammation when the immune system is underactive and thus damage the intestinal epithelium, allowing endotoxins to enter the bloodstream, with the consequence of systemic endotoxemia. During the development of endotoxemia, LPSs bind to toll-like receptor 4 (TLR4) and increase the concentration of inflammatory markers such as IL-1α, IL-6, INF-γ, and TNF-α (38), and consequently systemic chronic intestinal inflammation may develop. Studies have shown that the removal of LPS from the circulation contributes to the recovery from intestinal inflammation (82). When this is impaired, these molecules enter the bloodstream where they activate inflammatory cytokines, with systemic inflammation as a consequence (62).

First, we tested whether the chosen flavonoids and LPSs cause a change in the viability of our cell cultures. We found that the treatments with quercetin (25, 50 μg/mL) could significantly enhance (*p* < 0.001) the metabolic activity of the cells. Otherwise, luteolin showed significant cytotoxic effects (*p* < 0.001) in the case of PBMC cultures when 25 and 50 μg/mL concentration treatments were used. Therefore, we also applied 12.5 μg/mL treatment concentrations, but in case of luteolin significant reduction (*p* < 0.001) in metabolic activity of cells was observed again. The reducing effect of luteolin on metabolic activity was also observed in primary hepatocyte cultures at 16 μg/mL concentrations using the CCK-8 assay (83). In further experiments we used concentrations of 25 and 50 μg/mL for the flavonoid substances based on their IC_50_ values and our experience on other cell culture experiments. Flavonoids and other antioxidant compounds are known to have not only antioxidant but also prooxidant effects which depends on the concentration of the substances and the ROS status of the environment (84). In our studies, the two applied concentrations (25, 50 μg/mL) of quercetin, luteolin and GSOP were able to decrease the LPS induced ROS significantly (*p* < 0.001), so only their antioxidant effects were observed. Antioxidant activity of quercetin is well described, it is able to protecting red blood cells from oxidative stress and also playing an important role in the binding of reactive oxygen derivatives and it possesses immunomodulatory activity against endotoxins (85). The free radical scavenging capacity is mainly due to the double bond formed between the hydroxyl groups, the second and third carbon atoms (7). While luteolin structurally belongs to the flavone subgroup, with an oxygen atom in one ring and a double bond between the second and third carbon atoms. Hydroxyl groups are also present in the compound and, together with the double bond between the second and third carbon atom, are responsible for the antioxidant activity, which can be exploited to combat oxidative stress, as luteolin is effective in neutralizing reactive oxygen derivatives. Its aglycone form is more biologically active than its glucoside form, a more potent antioxidant and anti-inflammatory (33). However, the structure of proanthocyanidins consists of polyhydroxyflavanol oligomers or polymers, which are flavonoid compounds. Polyhydroxyflavanol polymers are able to participate in the fight against pathogens, the degree of polymerization determining the bioavailability. Procyanidine trimers and dimers have been shown to be highly resistant to pathogens in the stomach and small intestine. The structure of proanthocyanidins in the grape seed contains fewer polymers than the proanthocyanidins in the skin, so they may be more effective for enteric diseases. Due to the o-diphenol groups, offer greater protection against oxidative stress than ß-carotene, vitamin C and E (30).

If PBMC are stimulated with polyclonal activators, they will produce different cytokines and activation markers. When using PBMC as a model of endotoxaemia, it should be taken into account when drawing the final conclusions that these blood-derived cells are different from cells derived from intestinal tissue and that they lack environmental stimulation under *in vitro* conditions (86). PBMC can be used to identify cytokines activated during inflammation, such as TNF-α and interleukin-17 (87), and thus can be used to detect endotoxin-induced processes. TNF-α is an inflammation-stimulating cytokine that plays a central role in the regulation of the systemic inflammatory response and is produced primarily by macrophages. In our investigation both LPS-induced increases in TNF-α were significantly attenuated, in the case of quercetin at 50 μg/mL and in the case of luteolin at 25 and 50 μg/mL which is consistent with the results reported in the literature. For instance, *Hippophaë rhamnoides* (sea buckthorn) contains quercetin among others. In the body, quercetin reduces the effects of inflammatory mediators and cytokines such as TNF-α, IL-6, thus exerting anti-inflammatory and anti-tumor effects (10). The extract from the plant can attenuate LPS release from Caco-2 cells and TNF-α secretion on PBMC and PMN cells (88). Luteolin induced fewer CD4+ IL-4 secreting cells on PBMC cells (89). In addition, in *E. coli* O26:B6 LPS induced RAW 264.7 macrophage cell lines, luteolin and quercetin have also been shown to inhibit TNF-α and IL-6 secretion (90), but phorbol 12-myristate 13-acetate (PMA) triggered TNF-α production was reduced by quercetin has also been demonstrated in primary equine-derived white blood cell cultures as well (91).

Although GSOP decreased ROS at both applied concentrations, at the at 25 μg/mL concentration it increased TNF-α secretion. Therefore, it can be concluded that the concentration of GSOP may play an important role in the secretion of certain proinflammatory cytokines. It is known that flavonoids like other antioxidant agents, can act as prooxidants. This effect is mostly associated with the applied concentration of antioxidants. Because of the above mentioned activity they can have unexpected effect and can cause oxidative damage (84) Prooxidant effect of GSOP may not only depend on the dose but also on the duration of administration. In other cases when GSOP is used in combination with other dietary components, they can also mitigate the antioxidant/prooxidant activities (92). Smaller fractions of proanthocyanindins (monomer-tetramer) decreased IL-1β expression of phytohemagglutinin (PHA)-stimulated cells however oligomers (pentamer-decamer) increased it. So may be also the size of the fractions can be a factor in influencing the inflammatory processes (93). In another study proanthocyanidin treatment at 25 μg/mL lead to significant reduction of IL-1β, IL-6, and TNF-α than control at 4 h after *E. coli* O111:B4 LPS challenge in PBMC from pigs (94). The difference between the results may be due to differences between the animal species or the sampling time. However TNF-α has a strong effect on the production of other cytokines including IL-10, so we conclude that if TNF-α changes, other cytokines will also change (95).

The *in vitro* effects of flavonoids have been investigated on a wide range of monolayer cell cultures, among intestinal epithelial cells, quercetin reduced ROS and IL-6 in IPEC-J2 cells (96), IL-2 (97) and inhibited TLR2-NF-kB pathway in human PBMCs (98). Luteolin and GSOP antioxidant and antibacterial effect was also demonstrated on IPEC-J2 cells (72). GSOP may contribute to the maintenance of intestinal health and canine IBD through influencing the gut microbiome and bile acid metabolism (99). Although the low bioavailability of the flavonoids may limit their *in vivo* efficacy, numerous studies are underway to improve their absorption and distribution (100). This poor utilization may be helped by nanoparticles (9), but it may also be that their local effects on the gut have enough positive effect and they may indirectly can influence systemic health through intestinal well-being.

Our results suggest the use of quercetin, luteolin and GSOP can be effective as adjuvant therapy in small animal medicine, mainly in chronic inflammatory processes sustained by endotoxemia, or in intestinal diseases with possible loss of intestinal barrier integrity or microbiome disruption. Further *in vitro* and *in vivo* studies are required prior to clinical use. Further experiments are underway to investigate flavonoids in more complex ways, including organoid model studies one of the most accurately *in vitro* model of the intestinal tract, and co-culture experiments to model the relationships between white blood cells and intestinal epithelial cells. Following *in vitro* experiments, the effects of *per os* flavonoid supplementation on the intestinal tract integrity and microbiome of dogs should be investigated in animal studies and the systemic effects of flavonoids should be tested. It is well known that most flavonoids are poorly absorbed from the intestinal tract, so their systemic effect may be less pronounced. Because of this it is important to determine whether flavonoids are able to mitigate the effects of chronic inflammation caused by endotoxemia *in vivo* and to define their exact dosage before clinical use. In addition to healthy canine models, experimental inclusion of dogs with chronic enteropathy may be warranted for these studies, as changes in integrity may also influence the possible systemic effects of flavonoids, It is hoped that the safe and effective use of flavonoids in dogs suffering from chronic enteropathy and its role in reducing stress caused by chronic endotoxemia can be established.

## Data Availability

The original contributions presented in the study are included in the article/supplementary material, further inquiries can be directed to the corresponding authors.
